# Variations in stability revealed by temporal asymmetries in contraction of phase space flow

**DOI:** 10.1038/s41598-021-84865-8

**Published:** 2021-03-11

**Authors:** Zachary C Williams, Dylan E McNamara

**Affiliations:** 1 Nicholas School of the Environment, Duke University, Durham, NC USA; 2 Department of Physics and Physical Oceanography, University of North Carolina, Wilmington, NC USA

**Keywords:** Statistical physics, thermodynamics and nonlinear dynamics, Scientific data

## Abstract

Empirical diagnosis of stability has received considerable attention, often focused on variance metrics for early warning signals of abrupt system change or delicate techniques measuring Lyapunov spectra. The theoretical foundation for the popular early warning signal approach has been limited to relatively simple system changes such as bifurcating fixed points where variability is extrinsic to the steady state. We offer a novel measurement of stability that applies in wide ranging systems that contain variability in both internal steady state dynamics and in response to external perturbations. Utilizing connections between stability, dissipation, and phase space flow, we show that stability correlates with temporal asymmetry in a measure of phase space flow contraction. Our method is general as it reveals stability variation independent of assumptions about the nature of system variability or attractor shape. After showing efficacy in a variety of model systems, we apply our technique for measuring stability to monthly returns of the S&P 500 index in the time periods surrounding the global stock market crash of October 1987. Market stability is shown to be higher in the several years preceding and subsequent to the 1987 market crash. We anticipate our technique will have wide applicability in climate, ecological, financial, and social systems where stability is a pressing concern.

## Introduction

Comparing stability across systems or forecasting a change in stability when underlying dynamical equations are not known is a central challenge throughout science^1^ with far reaching societal relevance. Despite this, there is a lack of an agreed upon interpretation of stability and how it is measured empirically. This is likely due to stability being considered across many disciplines and in a broad array of systems, from simple bifurcating population models to climate models with many nonlinear, interacting parts. We present a coherent and unified tool set for gaining insight into stability based on nonlinear dynamical systems theory, which contains the theoretical apparatus to understand stability in a wide range of contexts.

A continuous-time autonomous dynamical system can be written as a set of *n* first-order ordinary differential equations ${\frac{{d {\vec{x}}}}{{dt}}} = {\vec{F}}({\vec{x}})$, where ${{\vec {x}}}=(x_1,x_2,\ldots ,x_n)$ are the *n* state-variables and ${{\vec {F}}}$ is a vector field governing their evolution. By projecting each variable onto its own axis, the evolution of the system forms a trajectory in the *n*-dimensional phase space. The basin of attraction is composed of all states which, through the action of dissipation, eventually lead to the attractor subset of the phase space. This can be described mathematically by tracking the evolution of many states initially contained within a *n*-dimensional volume *V*(*t*):

1 $$V(t) = V(0)e^{{\Lambda t}}$$

where $\Lambda$ is the phase space volume contraction rate. For dissipative systems it is always the case that $V(t)<V(0)$ and so $\Lambda <0$. Analytically $\Lambda$ can be obtained by taking the divergence of the vector field $\Lambda =\nabla \cdot {{\vec {F}}}$. Stability in this setting is conditioned on two properties of a system’s phase space; the basin of attraction and dissipation^2^. Stability decreases when either the attractor basin range is diminished relative to the size of external system perturbations or the amount of dissipation in the dynamics is reduced. With respect to Eq. (1), a reduction in dissipation corresponds to a decrease in the magnitude of $\Lambda$.

Most previous work exploring stability use metrics that capture time series variations and claim to provide a warning of looming change in a system’s attractor^3–5^. These methods fall under the umbrella of “early warning signals” for so called critical thresholds. The most prominent of these techniques is referred to as critical slowing down (CSD)^4^. CSD indicators such as increasing autocorrelation and variance in state variables tend to rise for some systems which presages a critical transition (or tipping point)^6^. While these methods purport to provide a prediction of looming system change, the essence of the technique is to measure system stability and then infer that a change is coming (but not say when it is coming or what amount of stability loss causes such a change). A strong limitation to these metrics, even when just being used as a stability indicator, is the built in assumption that the dynamics are dominated by the return of a system to a fixed point after a perturbation and that internal system variability remains constant as stability changes. This assumption breaks down for even modest increases in system complexity where variance and autocorrelation can be tied to intrinsic system dynamics and indeed many previous studies have detailed examples where CSD metrics do not provide insight into system stability^7–11^.

A more recent effort to quantify stability uses time series of multiple ecological species interacting in a network^12^. This novel approach uses Convergent Cross Mapping (CCM) to identify coupled species and then builds a linear prediction model from the reconstructed phase space. While this improves upon previous efforts that focus on simple systems, the appeal to linearized stability metrics keeps the focus of the analysis on a system’s return from small perturbations in a linear setting. Said another way, the full scope of potential sources of nonlinear variability and their relation to a system’s stability is neglected.

A critical component of this study is how dissipation, revealed in a system’s phase space behavior, is manifested on and near the attractor and how it can be revealed using only time series data of the state variables. In this way, we are not assuming a priori that stability is only revealed in how a system responds to perturbations nor are we assuming any particular type of attractor change such as a simple fixed point bifurcation. Our approach is much more general.

Dissipation arises when differences in state variables are diffusively damped, mixed, or reduced in the phase space and as such it is directly related to the global phase space volume contraction rate and is inversely related to time of decay to an attractor^2^ (hereafter referred to as volume contraction rate). Consequently, systems with more dissipation are more stable; as a system is drawn more rapidly toward the attractor, state trajectories are more likely than not to stay near to the attractor in the future. Measuring the decay time to the attractor is difficult as it is rare that an observer can conclude precisely when a system is outside its attractor. Additionally, dissipation is not trivial to measure when the system is inside the attractor. Consider the simple cases of a fixed point and limit cycle. For these systems, the evolution appears conservative on the attractor because there is zero net convergence and divergence. The dissipation in these cases is removing energy injected from outside the system (in the form of forcing), and so measuring dissipation is difficult because states are no longer converging on the attractor. In the more complicated setting of strange attractors, convergence and divergence occur simultaneously and heterogeneously throughout the attractor^13,14^, in contrast to fixed points or limit cycles. The divergence is related to sensitivity to initial conditions, and convergence (dissipation) acts to keep the systems constrained into a fixed attractor volume. Despite these apparent difficulties in measuring dissipation, we put forth an empirical technique and associated metric that provides a direct correlation to the amount of dissipation in a system. Dissipation as referred to throughout this manuscript is synonymous with phase space volume contraction, e.g. higher rate of dissipation in system dynamics implies higher volume contraction rate in the phase space, and hence more stability.

The classic way to measure the tendency of trajectories to both expand and contract in phase space is by determining the Lyapunov exponents^15,16^. Consider two points in phase space that are initially near neighbors at time *t*: $\left\Vert {{\vec {x}}}_1(t)-{{\vec {x}}}_2(t)\right\Vert \ll 1$. After *L* time has passed, the distance between these points may grow or shrink approximately as $\left\Vert {{\vec {x}}}_1(t+L)-{{\vec {x}}}_2(t+L)\right\Vert = \left\Vert {{\vec {x}}}_1(t)-{{\vec {x}}}_2(t)\right\Vert e^{\sigma _1 L}$. Inverting to solve for the growth rate yields the Maximal or Largest Lyapunov Exponent ($\sigma _1$):

2 $$\sigma _{1} = \mathop {\lim }\limits_{{L \to \infty }} \mathop {\lim }\limits_{{\left\| {{\vec{x}}_{1} (t) - {\vec{x}}_{2} (t)} \right\| \to 0}} {\frac{1}{L}}\ln \left( {{\frac{{\left\| {{\vec{x}}_{1} (t + L) - {\vec{x}}_{2} (t + L)} \right\|}}{{\left\| {{\vec{x}}_{1} (t) - {\vec{x}}_{2} (t)} \right\|}}}} \right)$$

If *L* is taken to infinity then $\sigma _1$ is referred to as global, and if it is evaluated over shorter times it is considered a finite or local LE and is usefully explored as a function of *L* and position in phase space (*t*). The maximal LE, $\sigma _1$ is diagnostic of the dynamics. For example, $\sigma _1>0$ is indicative of chaos (i.e. sensitivity to initial conditions) while $\sigma _1<0$ is indicative of fixed point dynamics. For a dissipative nonlinear dynamical system with *n*-degrees of freedom there are *n* Lyapunov exponents constituting a spectrum of LE ($\sigma _1,\ldots ,\sigma _n$). The global spectrum of LEs is an invariant of the system and for chaotic attractors there is at least one positive (nonlinearity) and one negative (dissipation) exponent. The sum of the spectrum of LEs is equal to the phase space volume contraction rate^2^, $\Lambda = \sum _{i=1}^{n} \sigma _i$. In theory, if the spectrum of global LEs can be determined, then the volume contraction rate, and therefore dissipation rate and stability, can be directly inferred. This is easily accomplished when the underlying evolution laws are known.

In time series applications where data is usually available for only one of many degrees of freedom, the Lyapunov spectrum can be obtained by first invoking Taken’s embedding theorem to reconstruct the attractor. Wolf’s algorithm, which tracks orbital stability^16^, can be applied to the reconstructed attractor to reveal the LE spectrum, however only the largest (positive) global LE is considered a reliable estimate^17,18^, and so the volume contraction rate is not reliably known. The efficacy of Wolf’s algorithm is limited by high sensitivity to noise, large data length requirements, choice of embedding dimension, and the strict requirements placed on identifying neighboring trajectories. Other techniques have also been proposed to uncover the full spectrum by considering the evolution of various types of perturbations, see^19^ for a review—needless to say, empirical settings almost never allow for one to explore ideal perturbation directions.

Other approaches for exploring phase space volume behavior involve measuring various entropy associated metrics. The Kolmogorov-Sinai (KS) entropy is related to the sum of positive Lyapunov exponents through Pesin’s identity^20^. The practical limitation of the KS entropy for empirical work is the strong reliance on the nature of how phase space is partitioned for the calculation. Other metrics such as permutation entropy^21^ are more easily computed however they lack a clear quantitative connection to KS entropy. Despite this, permutation entropy has been shown to provide utility in indicating when a system has crossed a bifurcation point^22^ but a definitive trend related to system stability has not been shown. A more recent advance based on a modified permutation entropy measure^23^ provides a reliable connection to KS entropy for systems with no noise present. This advance helps one to distinguish between chaotic and stochastic system dynamics^24–26^ but again a relationship between this modified permutation entropy to the system stability is not clear. Other recent advances exploring systematically perturbed systems explicitly connect entropy-production and phase space volume contraction for nonequilibrium systems^27^. Additionally, the Crooks Fluctuation Theorem^28,29^ connects the forward and reverse time phase space trajectories to dissipation. These approaches however admittedly do not apply for most realistic systems where the Lyapunov exponents and contraction rates fluctuate along the attractor. Our work builds on these previous advances by noting that since temporal irreversibility is due to entropy production (dissipation), and entropy production rate is equivalent to the phase space volume contraction rate, then a measurement associated with time asymmetry should reflect aspects of system stability.

## Phase space stability technique

To the best of our knowledge, no empirical technique currently exists to assess stability that does not presuppose linearity in the system structure or external perturbation response, require prohibitively large or noise free data with a minimal number of tunable parameters, have a theoretically justified connection to the origin of stability, or promise broad applicably across disparate systems. In this spirit, we hypothesize a measurement of stability based on phase space dissipation can be illustrated through temporal asymmetry in the aggregate phase space flow behavior. More specifically, our measurement technique probes how fast the average of all trajectories in phase space converge when proceeding forward in time and diverge backward in time and evaluates the difference. We show numerically that the magnitude of temporal asymmetry correlates with the amount of dissipation in the system, a quality of dynamics that unambiguously defines system stability.

Lyapunov exponents are an attractive approach to assess stability because of their generality, limited system assumptions, and clear connection to dissipation. Yet in empirical settings, one rarely has access to all perturbation directions in phase space. Moreover the distribution of local Lyapunov exponents around an attractor is typically not Gaussian^30^. In many circumstances it is not practicable to numerically evaluate the dissipation rate (volume contraction rate) via the sum of the globally averaged local LEs. Instead we describe a metric which correlates with dissipation rate that is based on the growth rate of the global average of local separation distances of initially nearby trajectories.

Consider two sets of phase space trajectories on the attractor each consisting of *N* states: ${\vec {x}}_1(t_i)={\vec {x}}_1(t_1), \dots , {\vec {x}}_1(t_N)$, and ${\vec {x}}_2(t_j)={\vec {x}}_2(t_1), \dots , {\vec {x}}_2(t_N)$. Pick a state with time index $t_i$ in ${\vec {x}}_1$ and find the state in $\vec {x_2}$ minimizing the Euclidean distance with respect to ${\vec {x}}_1(t_i)$. If this state is at time $t_{k(i)}$ in ${\vec {x}}_2$, then ${\vec {x}}_1(t_i)$ and ${\vec {x}}_2(t_{k(i)})$ are said to be nearest-neighbors. The distance between these two states for all later times is $d_{i,k(i)}(n):=\left\Vert {\vec {x}}_1(t_{i+n})-{\vec {x}}_2(t_{k(i)+n})\right\Vert$, where *n* is the number of time steps forward. Conversely, following the separation backwards in time is $d_{i,k(i)}(-n)$.

The first step toward our metric is to obtain the average of $d_{i,k(i)}(n)$ for many or all points in ${\vec {x}}_1$, where each time series of separation distances $d_{i,k(i)}(n)$ is normalized by the initial (i.e. closest) separating distance $d_{i,k(i)}(0)$ before averaging across $t_i$. To characterize the total quantity of flow convergence occurring heterogeneously across the attractor, we estimate the rate of growth from the phase-space averaged separation distances ($\lambda (L_n)$):

3 $$\lambda (L_{n} ) = {\frac{1}{{|L_{n} |}}}log\left( {{\frac{1}{N}}\sum\limits_{{i = 1}}^{N} {{\frac{{d_{{i,k(i)}} (n)}}{{d_{{i,k(i)}} (0)}}}} } \right)$$

where $L_n=n\Delta t$ is the time horizon and $\Delta t$ is the measurement time interval. The time horizon considered ranges from $n=0$ to $n=M$, where *M* is the number of time steps necessary to traverse a distance comparable to the average attractor size. We refer to Eq. (3) hereafter as the forward divergence rate $\lambda ^+:=\lambda (L_{+n})$. Similarly, if *n* is replaced with $-n$ in Eq. (3), the backward-time divergence rate is obtained, $\lambda ^-:=\lambda (L_{-n})$. As has been noted^31^ asymmetries exist when looking at phase space distance behavior forward and backward in time. Our purpose here is to assess the temporal asymmetry between a measure of average phase flow behavior looking forward in time and backward in time. This is achieved by differencing the backward and forward divergence rates. Finally our metric, symbolized as $\Delta \lambda$, equals the maximum of this difference:

4 $$\begin{aligned} \Delta \lambda =max\{\lambda ^--\lambda ^+\},\end{aligned}$$

We will show numerically for several model nonlinear dissipative systems, that the metric presented herein as Eq. (4) correlates with the dissipation rate allowing for an empirical estimate of system stability.

To be clear, the distinction with our approach and a similar metric using the largest Lyapunov exponent is that we have taken the average operation inside the logarithm to focus the calculation on phase space distance separations rather than exponential constants. Also, rather than only observe *L* at large times we are evaluating the distance separations over small *L* and finding when the asymmetry in time is maximal. The change in distance between nearby trajectories is known to have three distinct regions: an initial alignment to the direction of largest growth with small change in separation; an exponential separation phase; a final, relatively constant distance at the scale of the attractor. By exploring the first two regions closely to find differences in behavior when going forward and backward in time we will demonstrate there is an observed correlation with the system’s dissipation rate in a range of systems.

Before proceeding to more complex examples, consider the case of an overdamped simple harmonic oscillator. In this case the simple two dimensional phase space for the system is marked by constant convergence everywhere in the space toward the origin. The rate of convergence is simply the dissipation in the system. Any two nearby points taken as initial conditions will exponentially relax towards each other at the dissipation rate. In this case $\lambda ^+$ will measure exactly the negative of the value of dissipation in the system. And similarly, when time is reversed, it is clear that $\lambda ^-$ will measure exponential explosion of the nearby points, which will be given by the positive value of dissipation. Hence, in this pedestrian example, $\Delta \lambda$ measures twice the amount of dissipation in the system. What makes this so simple of course is that a perturbation from any given point to a neighbor point in the space, no matter the direction of the perturbation, is along the axis of the only convergence rate, or Lyapunov exponent, in the system. When one probes in varying directions within a higher dimensional space holding more complex dynamics, the quantitative match to dissipation is less direct. So while we do not expect $\Delta \lambda$ to measure exactly twice the dissipation in more complex dynamical systems, we will show that it nonetheless correlates with amount of dissipation and therefore stability in a range of systems.

## Results

The efficacy of our phase space stability technique is demonstrated by application to the canonical Lorenz system^32^. Utility is then demonstrated in reconstructed phase space of the Lorenz system, the Lorenz system with multiplicative noise, and the Rössler system with multiplicative noise. Finally we present an application to the financial market crash of October 1987, an event that is widely reported as having resulted from a reduction in internal system stability.

### Application to the Lorenz system

**Figure 1:**
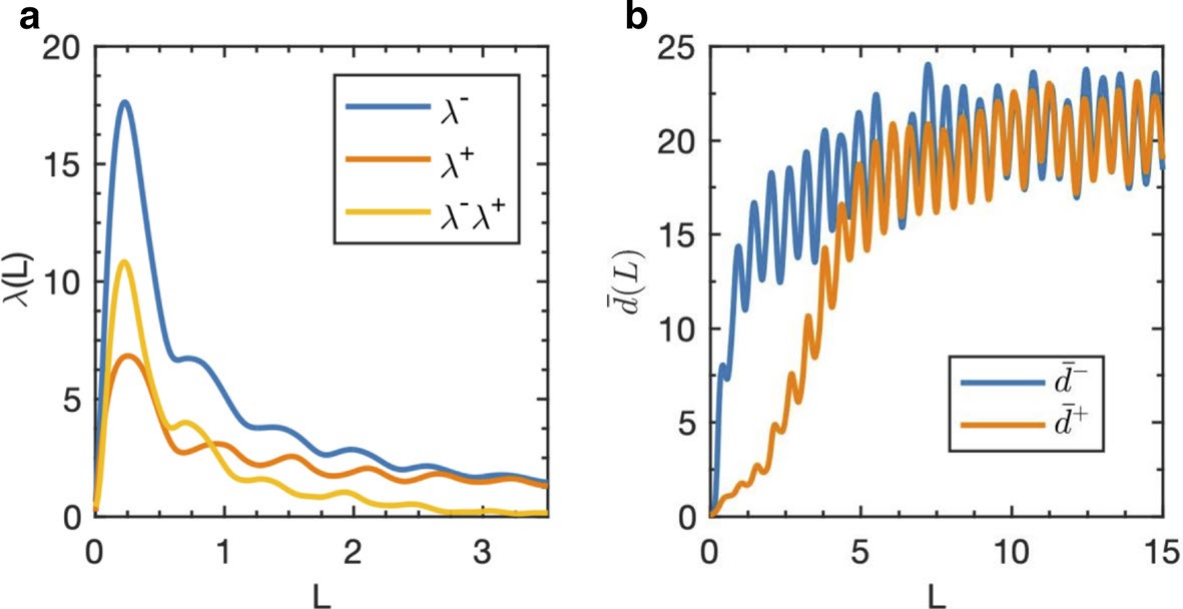
For the Lorenz system with parameters $r=45$, $b=8/3$, and $s=20$, panel (**a**) shows the rate of exponential divergence of the average separation distances as a function of time horizon ($\lambda (L)$). The forward time divergence rate ($\lambda ^+$) is orange, and the backward time divergence rate ($\lambda ^-$) is blue. The difference between backward and forward divergence rates is yellow. In panel (**b**) the average separation average across the phase space (the average of $d_{i,k(i)}$ over index *i* in Eq. 3) is shown forward in time (orange), and backward in time (blue).

The phase space stability technique is demonstrated first by application to the Lorenz system. Library and test sets are constructed from the three system variables *x*, *y* and *z*, and the parameter values used are $r=45, b=8/3$ and $s=20$. Library and test sets consist of 3000 points each. Details of numerical method are found in “Methods” section. The forward divergence rate $\lambda ^+$ as a function of *L* is the orange curve in Fig. 1a. When time is reversed, the divergence rate ($\lambda ^-$) results in the blue curve in Fig. 1a. The difference between backward and forward divergence rates is the yellow curve in Fig.  1a, which typically peaks at intermediate values of *L* before vanishing as *L* grows large. We note that in some cases there is no observable peak in $\lambda ^+$ or $\lambda ^-$, such that divergence rates are large for small *L* and decreasing with increasing *L*. In these cases, there is still a clear peak in their difference. Figure 1b shows the average separation distance (i.e. the phase space average in Eq. (3)) for forward and back backward time directions and illustrates the more rapid backward trajectory divergence suggested by the larger peak in Fig.  1a.

**Figure 2:**
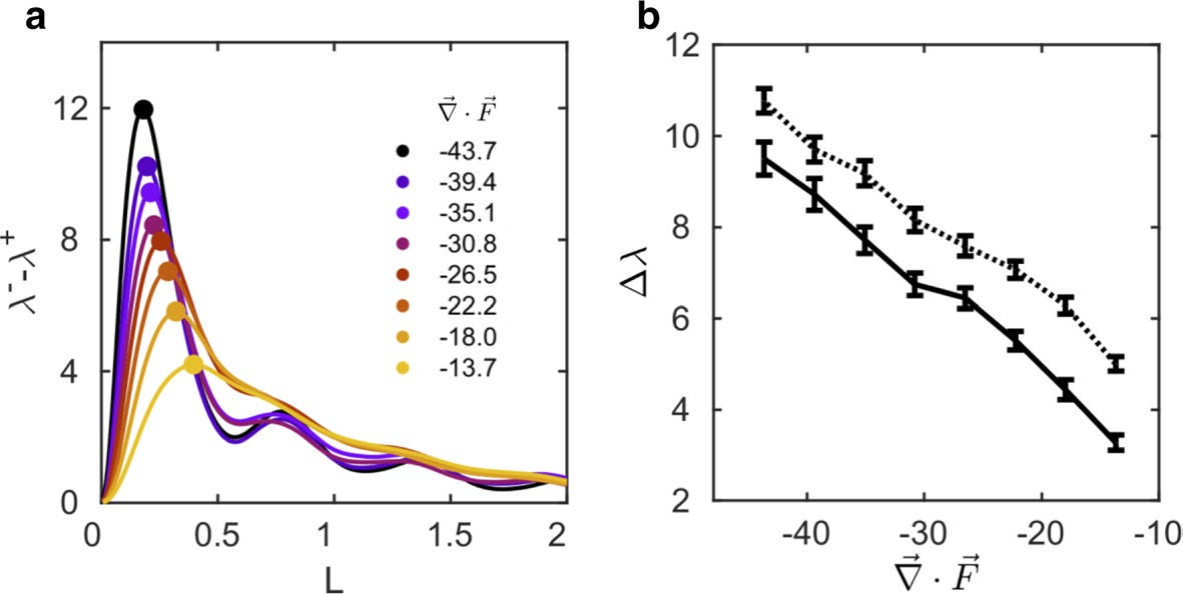
Variation of the stability metric with volume contraction for the Lorenz system. Panel (**a**) plots the difference between backward and forward divergence rates as a function of *L* based on the full Lorenz attractor where line color corresponds to the value of phase space volume contraction rate ${\vec {\nabla }}\cdot {\vec {F}}$ which is varied through the dissipative control parameter *s*. Panel (**b**) shows $\Delta \lambda$ as a function of volume contraction rate for the full Lorenz attractor (black), and the reconstructed attractor (dashed). The standard deviation of $\Delta \lambda$ is indicated by the error bars.

Next we show how $\Delta \lambda$ varies with the phase space volume contraction rate in the Lorenz system, where the contraction rate is controlled via the parameter *s*, that is ${\vec {\nabla }}\cdot {\vec {F}}(s)$. The parameter *s* is varied between between 10 and 40, while $r=45$ and $b=8/3$ are fixed. Figure 2a plots $\lambda ^-(L)-\lambda ^+(L)$ for 8 values of the contraction rate. A key element of Fig.  2a is demonstrated more clearly by the black curve in Fig.  2b where the peaks ($\Delta \lambda$) identified in Fig.  2a are plotted against volume contraction rate. Each point along the black curve in Fig.  2b corresponds to the ensemble mean of $\Delta \lambda$ pertaining to 100 repeated solutions of the Lorenz system with random initial conditions, and the error bars correspond to the ensemble standard deviation. A monotonic relationship between $\Delta \lambda$ and contraction rate is observed such that as the volume contraction rate increases (or stability increases), the magnitude of the asymmetry between forward and backward divergence rates is larger. An analytical representation of the non-isotropic flow divergence on the attractor and its variation with control parameters (here *s*) related to volume contraction escapes us and as far as we can tell, has escaped the community. Thus, as a first step, we are only revealing the correlation between our measure of the asymmetry $\Delta \lambda$ and the volume contraction rate. Additionally, if contraction rate were instead varied as a function of *b*, similar results are obtained (see Supplementary Figure 1 in the accompanying Supplementary Information file). Further, the parameter *r* can be changed without impacting the contraction rate and in this case $\Delta \lambda$ does not reveal a trend (see Supplementary Figure 2 in the accompanying Supplementary Information file).

To test the efficacy of the $\Delta \lambda$ metric in correlating with volume contraction rate for a reconstructed attractor, we reconstructed the phase space of the Lorenz system based on time series of the *x*-variable over the same range of control parameters used to generate the black curve in Fig.  2b. Both the library and test attractors consist of 3000 points, of which 1500 points were queried for calculation of $\Delta \lambda$. The dashed curve in Fig.  2b displays $\Delta \lambda$ as a function of volume contraction rate for 100 simulations per choice of *s*. Even in the reconstructed phase space, a statistically monotonic relationship between $\Delta \lambda$ and volume contraction rate is observed. This relationship holds even when overembedding the reconstructed attractor into four, five, and six dimensions (Supplementary Figure 3).

Importantly, if one were to only calculate the maximal global LE for the varying amounts of volume contraction shown, the correlation we observe vanishes (Supplementary Figure 4). Using only the maximum of the forward local LE, it is possible to see a trend with volume contraction, however this trend is not a reliable measure as it appears even when using phase randomized surrogate data, while our metric $\Delta \lambda$ does not (Supplementary Figure 5). We have observed that the relationship with volume contraction is weakly observable in the $2^{nd}$ and $3^{rd}$ moment distributions of the largest local LE and error growth rates (as defined in^13,19^ respectively). However we do not find this to be a particularly fruitful path for analysis since the connection between the distributional properties and the property of increased global volume contraction are not well understood. In the supplemental section, we present additional results using $\Delta \lambda$ as a measure of volume contraction in a system comprised of two coupled diffusionless Lorenz systems (Supplementary Figure 6). In this case the phase space dimension is six and there can be multiple positive and negative LEs. Here again, $\Delta \lambda$ tracks with the analytically determined volume contraction rate.

### Application to stochastic nonlinear dissipative systems

While the Lorenz system is useful for illustrative purposes, it is rare that an empirical investigation will find such a smooth, low dimensional dynamical system. More commonly, irregular system behavior is driven by both low dimensional nonlinearity and the influence of noise, which can be interpreted as a connection to a large reservoir of unmeasured degrees of freedom. To explore the efficacy of our metric in measuring stability when noise is dynamically embedded into the low dimensional nonlinear dynamics, we investigate the Lorenz system and Rössler system with multiplicative (state-dependent) Gaussian noise. See the supplemental section for an analysis of the Lorenz system with observational noise (Supplementary Figure 7).

**Figure 3:**
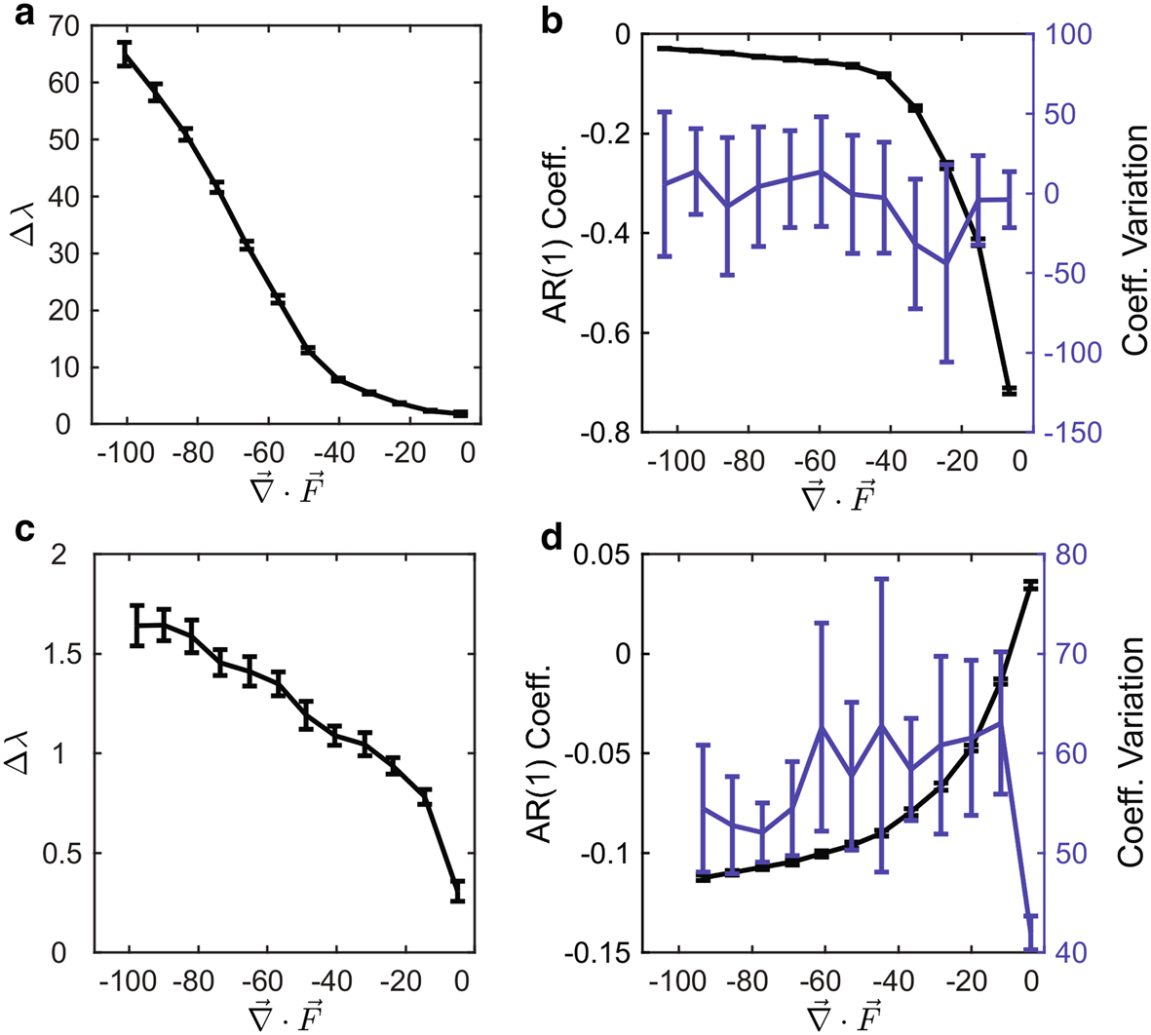
$\Delta \lambda$ as a function of phase volume contraction rate for the reconstructed stochastic Lorenz attractor with linear multiplicative noise (**a**) and for the reconstructed stochastic Rössler attractor with multiplicative noise (**c**). Panels (**b**,**d**) compare the two most common critical slowing indicators: the Autoregressive lag-1 (AR1) parameter and coefficient of variation corresponding to the Lorenz (**b**) and Rössler (**d**) systems with multiplicative noise respectively, given as a function of phase volume contraction rate.

The variation of $\Delta \lambda$ as a function of contraction rate for the reconstructed phase spaces of the stochastic Lorenz and Rössler systems is shown in Fig.  3a,c respectively. The error bars correspond to the ensemble mean and standard deviation from 100 repeated solutions of both systems with random initial conditions. In both systems, $\Delta \lambda$ increases as the volume contraction rate increases.

For systems that approach a simple bifurcation when varying a control parameter, the coefficient of variation and autoregressive lag-1 coefficient, *AR*(1), have been demonstrated to increase thereby providing an indication of decreasing stability. These measures are commonly referred to as critical slowing down indicators. For the stochastic Lorenz and Rössler systems, the coefficient of variation and *AR*(1) coefficient as a function of volume contraction rate are shown in Fig.  3b,d. The coefficient of variation for the stochastic Lorenz system is the blue line in Fig.  3b and does not bear any relation to the volume contraction rate. The *AR*(1) coefficient (black line) in Fig.  3b is negative while decreases in magnitude as the volume contraction rate becomes larger. In the framework of critical slowing down, a system that is losing stability should display an increase in the lag-1 autocorrelation, however the opposite appears to be the case for the stochastic Lorenz system. Turning to the stochastic Rössler system, the coefficient of variation is the blue line in Fig.  3d and there again appears to be no dependence on volume contraction rate. The *AR*(1) coefficient (black line in Fig.  3d increases with decreasing volume contraction rate. In this case the *AR*(1) coefficient is in agreement with $\Delta \lambda$ (Fig. 3c), where both metrics indicate stability is decreasing as the volume contraction rate grows smaller. Last we performed a surrogate data test of $\Delta \lambda$, *AR*(1), and the coefficient of variation for the Rössler system as a function of volume contraction rate shown in Supplementary Figure 8. The *AR*(1) coefficient reproduces the same signal as in Fig.  3d even after the effective removal of dynamics via fourier phase randomization (see “Methods”). In contrast, $\Delta \lambda$ shows no variation with volume contraction when surrogate data are used. This further highlights the strength of $\Delta \lambda$ as a measurement of stability based on the system dynamics.

We explored the performance of $\Delta \lambda$ as a function of the test and library set lengths used to reconstruct the attractor in phase space, and as a function of the number of points evaluated within the test set (*N* from Eq. 3). Results presented in Supplementary Figure 9 pertain to the stochastic Lorenz system and shows increasing time series length allows for finer variations in volume contraction rate to be discerned. We note that there is no theoretically justified universal data length requirement for attractor reconstruction and data length requirements may be unique to each system. However it is safe to assume that as the dimensionality of a system increases, more data is needed to faithfully reconstruct an attractor.

### Application to stock market stability

Empirical analysis tools related to dissipative nonlinear dynamical systems^25,33^, and in some cases specifically attractor reconstruction^34,35^, have been used in a wide range of economic settings. In this spirit, here we apply our metric to evaluate financial market stability during the 1980s, with specific focus on time period surrounding the October 1987 financial market crash referred to as Black Monday. Financial markets can be considered as complex adaptive systems composed of many heterogeneous interacting agents who process information to form expectations based on exogenous (e.g. news) and endogenous sources (e.g. other agent opinions) with the goal of maximizing stock market investments^36,37^. Both empirical and theoretical studies show strong support for this dynamical systems conceptualization of economies and markets, for example see^33,38,39^. A market crash can result from both exogenous shocks to the economy (e.g. a pandemic) or endogenous dynamics (e.g. speculative bubbles), or some combination therein^40^. Black Monday was the single largest proportional drop in the history of the S&P500 and is considered to be entirely the result of internal dynamics, specifically positive feedbacks between speculative and fundamentalist stock traders^38,39^.

**Figure 4:**
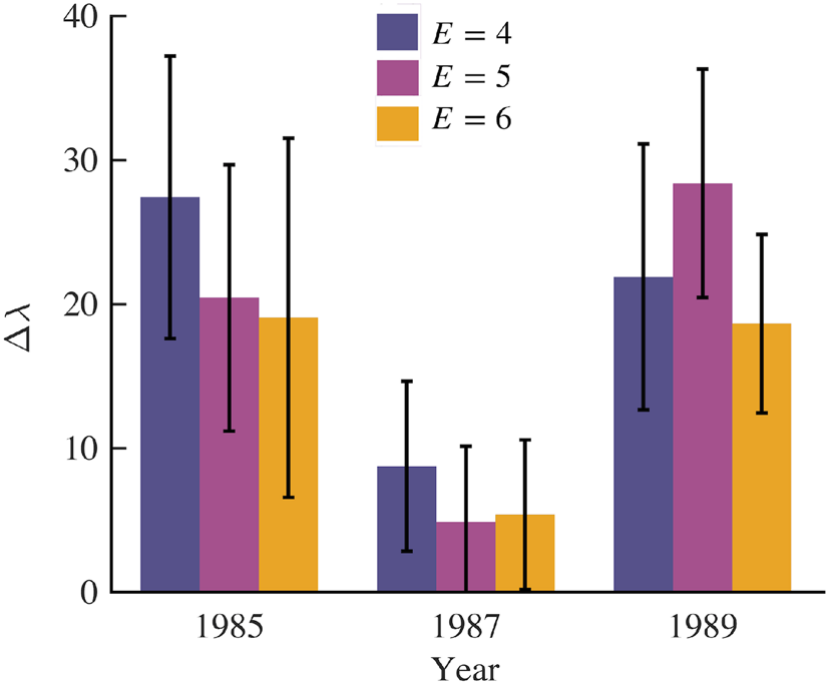
Stability of the S&P 500 index during three time periods in the mid to late 1980s. $\Delta \lambda$ is shown as a function of time (year) and for three values of phase space embedding dimensions (indicated by color). Error bars correspond to one standard deviation of the distribution of $\Delta \lambda$ calculated over each corresponding time period. These results demonstrate that market stability was significantly lower in the time around the October 1987 global stock market crash, when compared to the years before and after the crash.

We apply the phase space stability technique to the price return time series of the S&P500. Figure 4 demonstrates that system stability $\Delta \lambda$ was higher in the years preceding and proceeding the 1987 crash, and was nearly absent in period around the crash. While previous work suggests a suitable embedding dimension ($D_E$)of 5 for the S&P 500 returns time series, robustness of the result is partially validated by testing $D_E$ of 4,5, and 6. For all three choices of $D_E$, $\Delta \lambda$ is lower in the time period around 1987.

## Discussion

We have presented a technique to quantify the phase space stability of nonlinear dissipative systems based on time series observations. The technique is applied in the context of canonical nonlinear and stochastic dynamical systems and we provide application to the S&P 500 time series that contains a verifiable financial market instability which occurred globally in 1987. Stability in the context of the present study refers to the internal system stability reflecting interplay of the underlying nonlinear and dissipative dynamic processes. Previous efforts to quantify stability from time series effectively assume stability is constant throughout the attractor and that a change in stability is immediately detectable. For example, Critical Slowing Down indicators would interpret a change in the amplitude or variability of an external stochastic forcing as a change in stability, even while nothing has changed in the internal dynamics. Our technique assesses the context of a perturbation (i.e. the attractor), and therefore would correctly predict no change in stability. This is because our technique quantifies the rate of dissipation across the whole attractor, as opposed to extrapolating local stability properties of the attractor which are well-known to vary heterogeneously throughout the attractor.

Why should we expect an observable temporal asymmetry in the phase space of dissipative nonlinear dynamical systems to exist and why would this be connected to stability? We hypothesize that backward time divergence is larger than forward time divergence owing to variations in the strength of converging and diverging regions on an attractor^19,41^ and the action of dissipation in reducing differences in system state. While volume contraction rate in the full space for the Lorenz attractor is constant, the rate of separation between trajectories varies around the attractor as the direction to near neighbors varies throughout the phase space. When choosing points to test for distance spreading, we use nearest neighbors which ensures that we have chosen from regions of strong dissipation and hence relatively strong flow convergence (dissipation reduces state differences). Conversely, when one marches backwards in time from these close neighbor points on the attractor, the flow tends toward divergence. To be clear, this is not true for every point used in the analysis but when averaged around the attractor, the choosing of very near neighbors has provided enough preference to areas of dissipation to reveal a strong time asymmetry. In fact, if one uses neighbors that are far apart to calculate $\Delta \lambda$, the asymmetry vanishes (not shown).

In the context of attractor reconstruction, the technique presented here is subject to the same limitations that have been carefully detailed elsewhere, e.g. data length and stationarity^17^. In application of our technique, a pre-analysis following the protocols outlined in^17^ should first be conducted to ensure that the reconstructed attractor reveals a signal of low dimensional nonlinear determinism. In this pre-analysis one may encounter spurious or irregular amounts nonlinear predictability as a function of embedding dimension, embedding lag, or prediction distance. This would be in principle due to insufficient data lengths, high-dimensionality, or the absence of nonlinear-determinism. Although it may not be possible to determine which is the cause. Another limitation is that a particular value of our dissipation metric, $\Delta \lambda$, does not always have an obvious connection to the analytically calculated rate of phase space convergence. Increasing the embedding dimension for the same system will decrease the magnitude of $\Delta \lambda$. That is to say $\Delta \lambda$ is itself a function of $d_E$ and it is the trend in $\Delta \lambda$ when $d_E$ is held fixed that is important. Said another way, it is only in comparing $\Delta \lambda$ for similar systems or evaluating $\Delta \lambda$ through time that one gains insight into relative stability. This caveat is equivalent to assuming the system under study is not changing in the number of effective degrees of freedom.

The range of potential applications for our dissipation metric is as wide as the range of utility for attractor reconstruction. One realm of application is in model testing. A given numerical model will have measurable and controllable amounts of dissipation. By comparing two simulations with varying amounts of dissipation to a time series from a natural system, one should be able to test a model’s ability to simulate the relative stability of the system in question by measuring our metric for the model and natural system. Beyond model testing, particularly provocative opportunities for using our metric include gaining insight into the amount of dissipation and stability and how that has changed over time in increasingly stressed climate, ecological, financial, or social systems.

## Methods

### Lorenz system

The Lorenz system is a set of coupled nonlinear ordinary differential equations. There are three degrees of freedom, *x*, *y*,  and *z*, and three constants *s*, *r*, and *b*.

5 $$\begin{aligned} {\frac{{dx}}{{dt}}} & = s(y - x) \\ {\frac{{dy}}{{dt}}} & = x(r - z) - y \\ {\frac{{dz}}{{dt}}} & = xy - bz \\ \end{aligned}$$

Numerical solutions are obtained using a fourth order Runge-Kutta method with time step $\Delta t=5\times 10^{-3}$. To ensure convergence onto the attractor, all numerical solutions were obtained after integrating for $5\times 10^4$ time steps. The phase volume contraction rate is obtained by taking the divergence of Eq. (5):

6 $$\begin{aligned} {\vec {\nabla }}\cdot {\vec {F}}&=-s-1-b \end{aligned}$$

The volume contraction rate is a function of two parameters *s* and *b*. For simplicity, we take *s* as the parameter controlling the divergence rate for all results pertaining to the Lorenz system. However we find similar results when taking *b* as the controlling parameter. Example time series of the *x* variable for the Lorenz system for various values of *s* are provided in Supplementary Figure 10.

### Stochastic Lorenz system

The Lorenz system with multiplicative noise is a set of Ito stochastic differential equations^42^:

7 $$\begin{aligned}{dx}&=s(y-x)dt+\sigma x\,dW_{t}\\ {dy}&=(rx-y-xz)dt+\sigma y\,dW_{t}\\ {dz}&=(xy-bz)dt+\sigma z\,dW_{t} \end{aligned}$$

The term $dW_t$ is the increment of a Wiener process that is independently drawn for each degree of freedom, and $\sigma ^2$ is variance. Parameter values for *r* and *b* are the same as those from Fig. 2. The volume contraction rate is similar to that in the deterministic Lorenz but with an additional term reflecting the contribution of the multiplicative noise term^43^:

8 $$\begin{aligned}{\vec {\nabla}}\cdot {{\vec {F}}}&=-s-1-b+3\sigma \lim _{t\rightarrow \infty }{\frac{W_t}{t}} \end{aligned}$$

The range of contraction rates in Fig.  3a is obtained by varying *s* between 2 and 100. The noise standard deviation ($\sigma$) is set to 0.2. Numerical solution of Eq. (7) is obtained using the Euler-Maruyama method with a time step of $\Delta t=5\times 10^{-3}$. The stochastic Lorenz attractor is reconstructed from the *x* variable using an embedding dimension $D_E=3$ and embedding time lag $\tau =20$. The multiplicative noise term contributes a small random component to the contraction rate (Eq. 8) and so ensemble results are displayed as binned averages. Example time series of the *x* variable for the stochastic Lorenz system for various values of *s* are provided in Supplementary Figure 11.

Performance of two common Critical Slowing Down indicators are examined for time series output of the stochastic Lorenz system (Eq. 7) as a function of volume contraction. To apply the *AR*(1) coefficient to the time series output of a continuous system, we first sample the time series data at an interval equal to the embedding lag ($\tau$, see “Attractor reconstruction”) used for delay embedding in attractor reconstruction. If this step is not taken, the *AR*(1) coefficient shows small variations occurring at the third decimal place, reflecting only the continuous time nature of the system. The *AR*(1) coefficient is estimated by taking the linear correlation coefficient of the time series and itself lagged at 1. The coefficient of variation is simply the standard deviation of the time series divided by the mean.

### Stochastic Rössler system

The stochastic Rössler system^44^ with multiplicative noise, similar to the stochastic Lorenz system (Eq. 7), is a set of Ito stochastic differential equations:

9 $$\begin{aligned}{dx}&=-(y+z)dt+\sigma x dW_t\\ {dy}&=(x+ay)dt+\sigma y dW_t\\ {dz}&=(b+xz-cz)dt+\sigma z dW_t \end{aligned}$$

where *a*,*b*, and *c* are parameters. The average volume contraction rate is:

10 $$\begin{aligned}{\vec {\nabla}}\cdot p {{\vec {F}}}=a-c+{\bar{x}}+3\sigma \lim _{t\rightarrow \infty }{\frac{W_t}{t}} \end{aligned}$$

Results presented in Fig.  3c are obtained by varying the dissipative control parameter *c* between 2 and 100, the noise standard deviation is $\sigma =0.2$, and the fixed parameter are $a=0.1$ and $b=0.3$. Numerical solution to Eq. (9) is obtained using the Euler-Maruyama method with a time step of $\Delta t=1\times 10^{-2}$. The stochastic Rössler attractor is reconstructed based on the *x* variable using an embedding dimension $D_E=3$ and embedding time lag $\tau =40$. Example time series of the *x* variable for the stochastic Rössler system for various values of *s* are provided in Supplementary Figure 12.

### Attractor reconstruction

The delay-embedding theorem offers a way to recover the complete phase space behavior of a dynamical system from a time series of just one of the system variables. To reconstruct the attractor, a time series, *x*(*n*), is embedded into a *d*-dimensional space to form a trajectory composed of vectors (${\vec {y}}_n$) whose components are lagged sequences of the original time series:

11 $$\begin{aligned} {\vec {y}}_n=[x(n),x(n-\tau ),\ldots ,x(n-(d-1)\tau )] \end{aligned}$$

where the constants *d* and $\tau$ are referred to as the embedding dimension and time delay respectively. Critically, the reconstructed attractor ${\vec {y}}_n$ is identical to the unknown attractor up to a smooth local change of coordinates, and contains all the topological properties of the unknown attractor i.e. system invariants.

There is an extensive literature on how to appropriately choose the values of $\tau$ and *d*. We take the first minimum of the mutual information^13^ to determine $\tau$ and the number of degrees of freedom from the originating system as the embedding dimension. When comparing stability between similar systems (as in Figs.  2,3, 4), the choice of $\tau$ is kept fixed. This is because an optimized prediction horizon is not the objective here. The objective is to detect relative changes in the flow contraction which could be obfuscated by embedding similar systems with widely varying embedding time lags $\tau$.

### S&P 500 index returns time series

Time series for S&P500^45^ returns are based on the adjusted closing prices *P*. The price return at time *t* over some interval *T* is:

12 $$\begin{aligned} r^T_t={\frac{P_t-P_{t-T}}{P_{t-T}}} \end{aligned}$$

For example when $T=1$ then the returns are daily. Since daily returns are very noisy, we analyze monthly returns ($T=20$) for the analysis presented herein. After obtaining monthly returns, the returns time series is divided into 3 groups spanning the years 1984–1986, 1986–1988, and 1988–1990. This way we test the stability preceding, during, and after the Black Monday crash of 1987. Within each group, we calculate $\Delta \lambda$ based on a sliding sliding window library and test set that are each 252 points (1 year). For example, in the grouping spanning 1984 through 1986, the first library set spans 01/03/1984 to 12/28/1984 and the test set spans 01/03/1985 through 12/28/1985 and $\Delta \lambda$ is estimated. This procedure is repeated by advancing to the start and end dates of both the library and test sets by 1 day, until the end date of the test set reaches 12/28/1986. The same procedure is applied to each group resulting in approximately 252 estimates of $\Delta \lambda$. The mean and standard deviation are presented in Fig.  4. The embedding time lag is obtained from the first minimum in the average mutual information from the returns time series spanning 1980-1990 and is found to be $\tau =10$. Previous studies have suggested an embedding dimension of around 5 for the S&P500^46^, therefore we present results corresponding to embedding dimensions of 4, 5, and 6.

## Supplementary Information

Supplementary Information

## Data Availability

MATLAB code available at https://github.com/zcwilliams/stability_metric.
