# Supplementary material for: Variations in stability revealed by temporal asymmetries in contraction of phase space flow
Source: Sci Rep. 2021 Mar 11;11:5730. doi: 10.1038/s41598-021-84865-8 (PMC7970983; doi:10.1038/s41598-021-84865-8)
Supplement: Supplementary file 1 — Supplementary Information [file 41598_2021_84865_MOESM1_ESM.pdf]

Supplementary Information For "Variations in stability revealed by temporal asymmetries in contraction of phase space flow"

Zachary C Williams[1,*] **and Dylan E McNamara**[2]

[1]Nicholas School of the Environment, Duke University, Durham, NC
[2]Department of Physics and Physical Oceanography, University of North Carolina, Wilmington, NC
[*]zachary.c.williams@duke.edu

Supplementary Figures

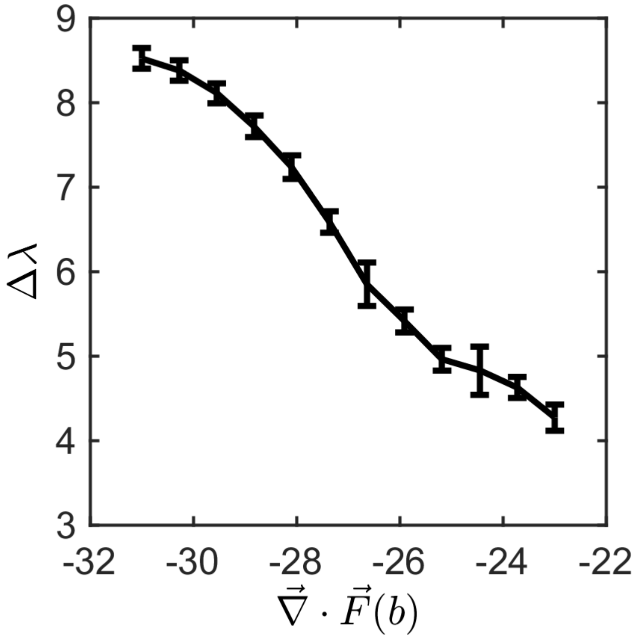

Supplemental Figure 1. The stability metric for the reconstructed Lorenz attractor with multiplicative noise ($\sigma = 0.2$). $\Delta\lambda$ is displayed as a function of the contraction rate, which is varied as a function of the parameter b between 2 and 10 (see Eq. 6 in main text). For each value of b, we compute $\Delta\lambda$ 100 times with random initial conditions, $r = 45$, and $s = 20$. Error bars are the 95% interval. The relationship here is commensurate with Figure 3a where the contraction rate is varied as a function of s.

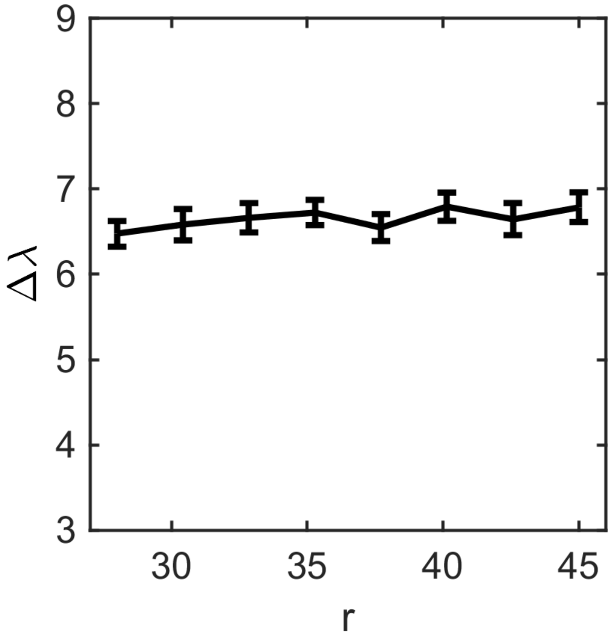

Supplemental Figure 2. The stability metric for the reconstructed Lorenz attractor with multiplicative noise ($\sigma = 0.2$). $\Delta\lambda$ is displayed as a function of the parameter r, with $s = 20$ and $b = 8/3$. Error bars are the 95% confidence interval. The volume contraction rate (Eq. 6 in text) is not a function of r and $\Delta\lambda$ does not vary in any significant manner.

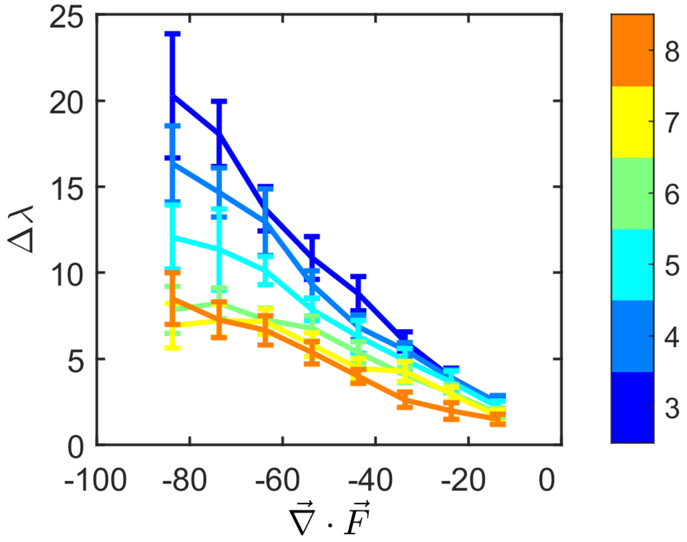

Supplemental Figure 3. For the reconstructed Lorenz attractor with multiplicative noise ($\sigma = 0.2$), $\Delta\lambda$ is displayed as a function of the volume contraction rate and the embedding dimension E which is indicated by the colorbar.

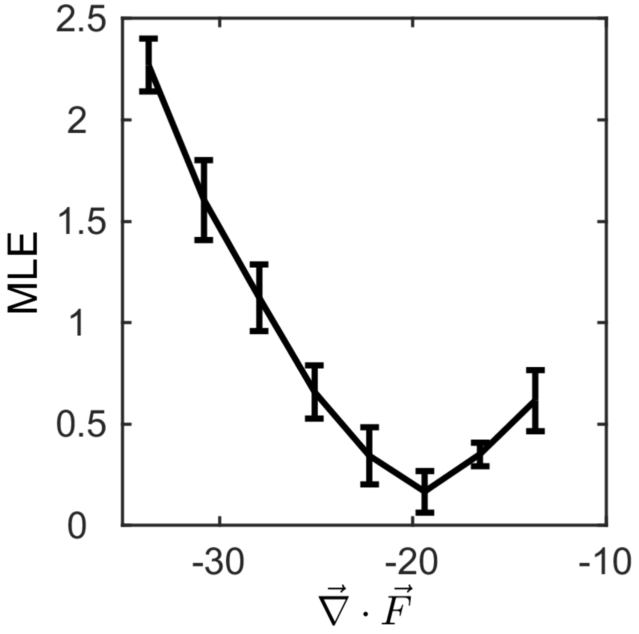

Supplemental Figure 4. For the reconstructed Lorenz system with multiplicative noise ($\sigma = 0.2$), the global maximum Lyapunov exponent (MLE) is shown as a function of the volume contraction rate, where s is varied from 10 to 30, $b = 8/3$, and $r = 45$. Time series consist of 18000 points. The MLE is the average value over the time horizon from $L = 30$ to 35. Error bars correspond to the 95% confidence interval based on 100 repeat simulations using time series with random intitial conditions.

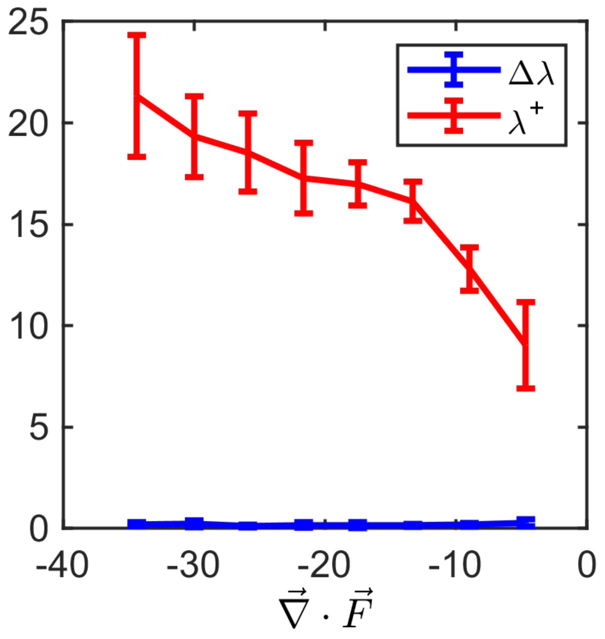

Supplemental Figure 5. Comparison of $\Delta\lambda$ and the maximum value of the forward time component of the stability metric λ^+ when applied to surrogate data generated from the Rössler attractor with a relatively low noise strength ($\sigma = 0.05$). The attractor reconstruction procedures follows the same protocol as in the text for the stochastic Rössler system. Surrogate data was generated following the Amplitude Adjusted Fourier Transform method described by Schreiber and Schmitz 1996.

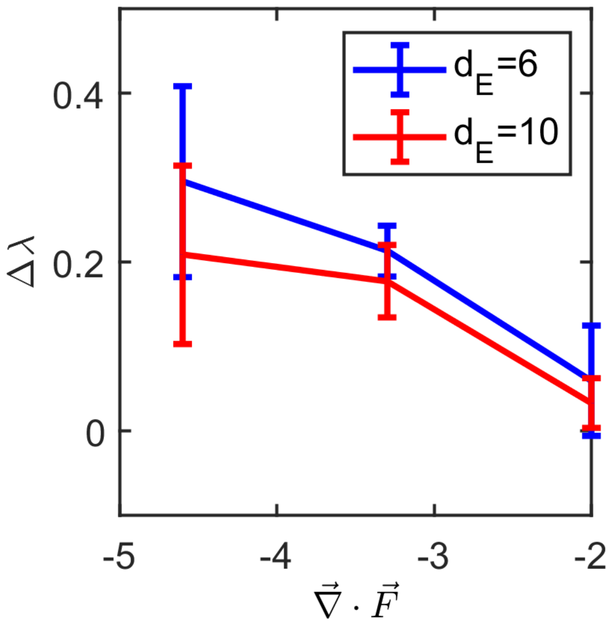

Supplemental Figure 6. The stability metric for a high-dimensional (>4) nonlinear dynamical system is demonstrated with the 6-dimensional coupled diffusionless Lorenz system (see Sprott 2010). Here, $\Delta\lambda$ is applied in the reconstructed phase space based on the the x variable. The blue line corresponds to an attractor embedding dimension (d_E) of 6, and the red line corresponds to an embedding dimension of 10.

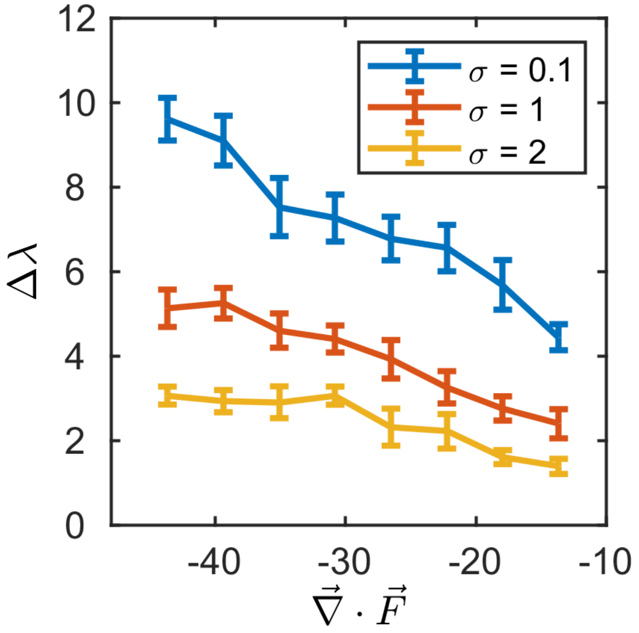

Supplemental Figure 7. For the reconstructed Lorenz attractor, $\Delta\lambda$ is displayed as a function of the volume contraction rate. Colors correspond to the varying levels of Gaussian noise contaminating the x variable prior to the attractor reconstruction procedure, where the standard deviation of the noise is σ.

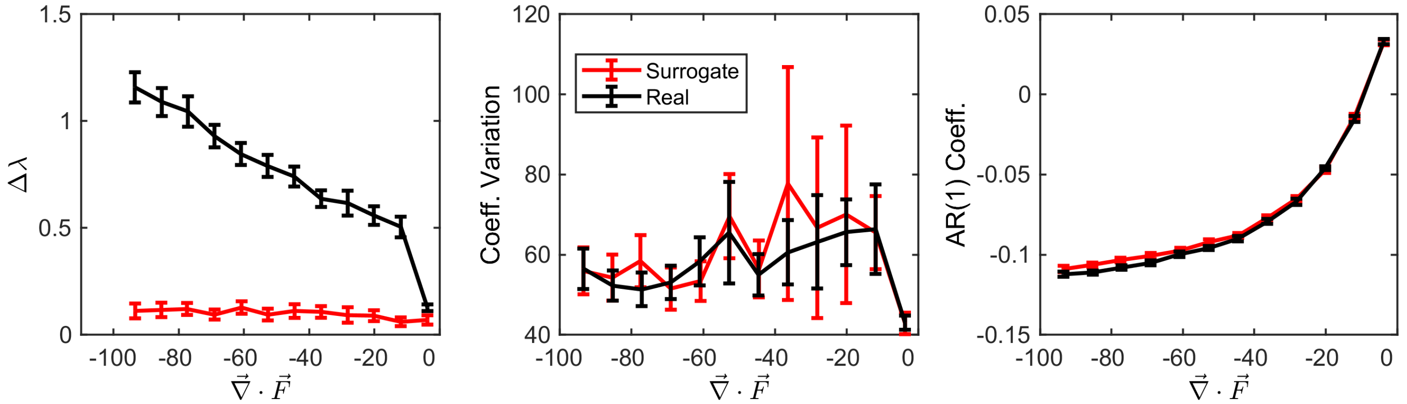

Supplemental Figure 8. Performance of $\Delta\lambda$ is compared with two common stability indicators for Critical Slowing Down for the stochastic Rössler system with multiplicative noise ($\sigma = 0.2$). The black line in each panel shows the metric as a function of volume contraction rate as applied to time series of x-variable in manuscript Eq. 9. The red line in each panel corresponds to the metric applied to surrogate times series for the stochastic Rössler system, where surrogates are created by the Amplitude Adjusted Fourier Transform method (Schreiber and Schmitz, 1996).

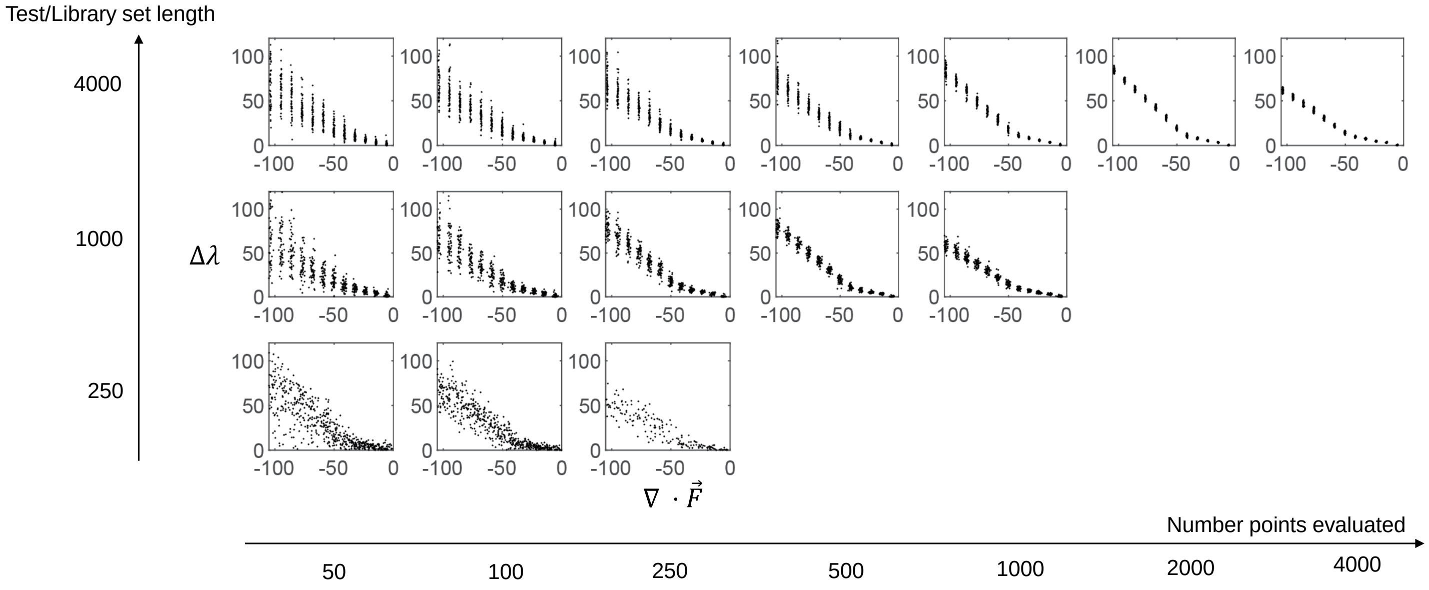

Supplemental Figure 9. For the reconstructed attractor of the stochastic Lorenz system with multiplicative noise ($\sigma = 0.2$), the relationship between $\Delta\lambda$ and volume contraction rate is illustrated for different time series lengths (vertical axis) and number of points evaluated for distance separation within the test set. For example a test set of length 4000 and where the number of points evaluated equals 2000 means exactly half of the available data were queried to evaluate λ^+ and λ^-.

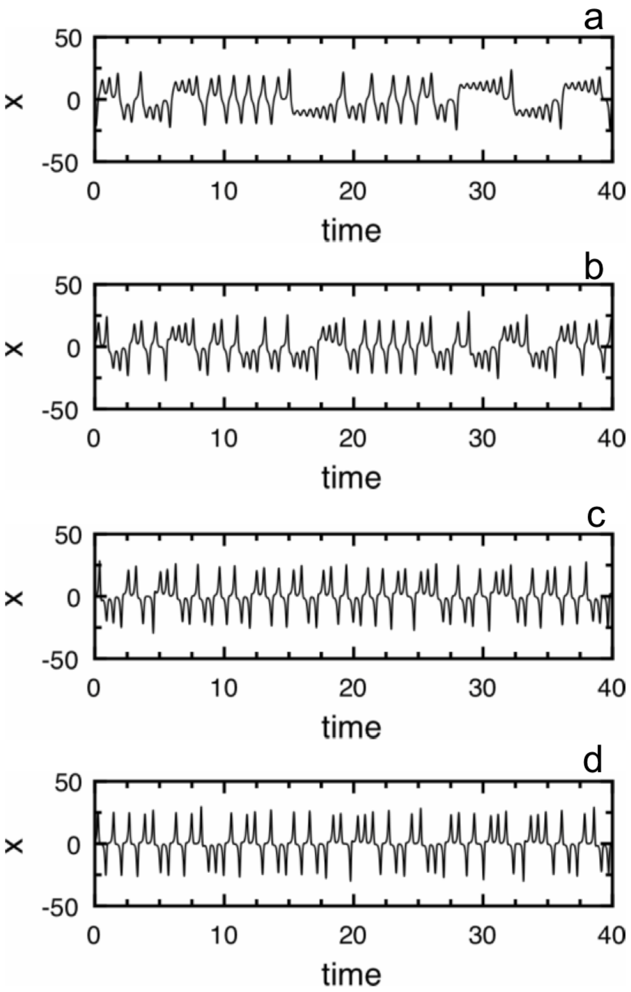

Supplemental Figure 10. Example of the x variable time series output for the Lorenz system corresponding to increasing values of the volume contraction rate (increasing a through d). Parameter values are $r = 45$, $b = 8/3$, and $s = 10, 20, 30$ and 40 corresponding to panels a,b,c, and d respectively.

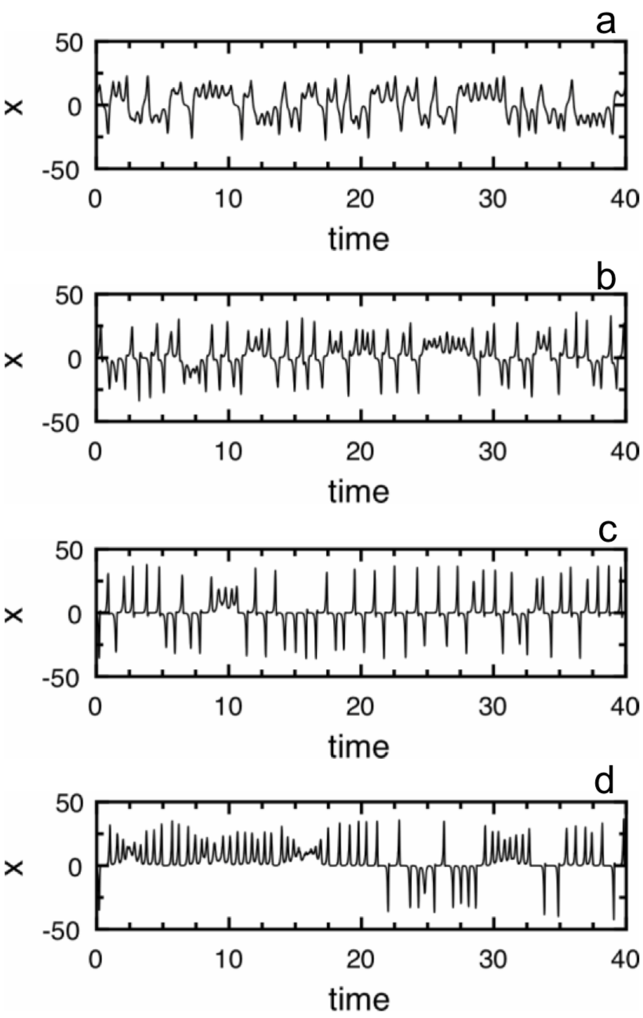

Supplemental Figure 11. Example of the x variable time series output for the Lorenz system with multiplicative noise ($\sigma = 2$) corresponding to increasing values of the volume contraction rate (increasing a through d). Parameter values are $r = 45$, $b = 8/3$, and $s = 10, 40, 70$ and 100 corresponding to panels a,b,c, and d respectively.

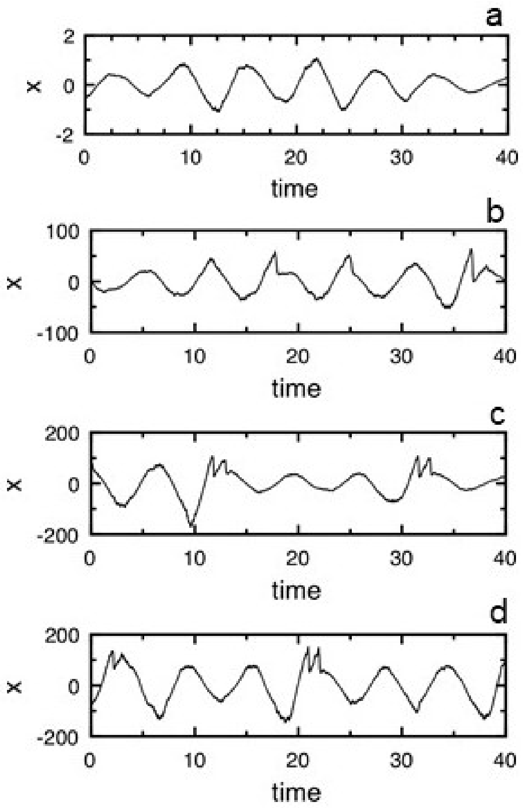

Supplemental Figure 12. Example of the x variable time series output for the Rössler system with multiplicative noise ($\sigma = 2$) corresponding to increasing values of the volume contraction rate (increasing a through d). Parameter values are $a = 0.1$, $b = 0.3$, and $c = 2, 35, 68$ and 100 corresponding to panels a,b,c, and d respectively.

References

Sprott, Julien C. Elegant chaos (2010): algebraically simple chaotic flows. World Scientific.
Schreiber T, Schmitz A (1996): Improved surrogate data for nonlinearity tests. Physical Review Letters 77: 635-638
